# A New Normal? Continued Impact of COVID19 on Alzheimer’s Clinical Trials Research

**DOI:** 10.1093/geroni/igab046.527

**Published:** 2021-12-17

**Authors:** Elizabeth Rhodus, Allison Gibson, Shoshana Bardach, Erin Abner, Gregory Jicha

**Affiliations:** 1 University of Kentucky, Lexington, Kentucky, United States; 2 University of Kentucky, University of Kentucky, Kentucky, United States; 3 Dartmouth University, Dartmouth University, New Hampshire, United States

## Abstract

The COVID-19 pandemic has led to unprecedented challenges in Alzheimer’s disease and related dementias (ADRD) clinical trials research. Scientists continue to grapple with the potential and multifaceted consequences of COVID-19. This presentation will discuss strategies used at a U.S. Alzheimer’s Disease Research Center to implement virtual methods to counter COVID-19’s impact on safety for continued research engagement; address the disparate impact by age, race, and ethnicity for online accessibility; and plans for virtual engagement in future research. As scientists navigate lasting implications of COVID-19, future study planning, design, and management will likely be altered. Specifically, increased awareness of participant-centered approaches, inclusion of psychosocial implications, and focus on ways to meet older adults’ unique needs of virtual accessibility will be needed. We must be intentional to counter COVID-19’s lasting impact on ADRD clinical trials research while maintaining rigor and reproducibility to uphold and progress advances toward treatment and cures for ADRD.

